# Assessing Clinical Frailty in an Emergency Department Context Using Electronic Health Records

**DOI:** 10.1093/geroni/igaf122.2363

**Published:** 2025-12-31

**Authors:** Jasmine Su, Ula Hwang, Natalia Sifnugel, Inessa Cohen, Debra Tomasino, Ling Hang

**Affiliations:** NYU Grossman School of Medicine, New York, New York, United States; NYU Grossman School of Medicine, New York, New York, United States; NYU Langone Medical Center, New York, New York, United States; Yale University, New Haven, Connecticut, United States; New York University, New York, New York, United States; Yale University, New Haven, Connecticut, United States

## Abstract

Frailty is a key predictor of health outcomes and mortality in older adults, yet physical assessments are time- and labor-intensive. The Claims Frailty Index (CFI) is a validated tool that estimates frailty with claims data, removing the need for physical assessments (Kim, 2017). However, its feasibility in emergency departments (EDs)—where older adults are frequent visitors—remains unclear. This study examines how frailty can be measured using electronic health records (EHR) from ED visits. We calculated CFI for 7,173 older adults (65+) who visited the ED at NYU Langone Health in December 2023, applying a one-year lookback of ICD-10 and CPT codes from prior encounters (PE) and historical diagnoses (problem list [PL], previous medical history [PMH]). The average CFI was low (0.121, SD ± 0.016), with the majority (95%) classified as non-frail (CFI< 0.15). Most historical diagnosis inputs came from PE (80%), while PMH (12%) and PL (16%) contributed less. Source-specific CFIs showed minimal variation: with a mean of 0.115 (SD ± 0.013) using only PE, 0.112 (SD ± 0.010) with PMH, and 0.109 (SD ± 0.012) with the PL alone. These findings align with prior research using inpatient and outpatient claims, which also reported low CFIs among older adults (CFI< 2.0). While CFI provides a scalable, data-driven approach to frailty assessment without requiring physical exams, its limitations include potential underestimation due to incomplete EHR documentation. Future research should refine CFI for ED settings and explore its use in predicting geriatric health outcomes, improving patient care, and enhancing ED decision-making.

